# Validation of an Asbestos Exposure Questionnaire (QEAS-7) for Clinical Practice

**DOI:** 10.3390/ijerph17249167

**Published:** 2020-12-08

**Authors:** Jaume Ferrer, Galo Granados, Santos Hernández, María-Jesús Cruz, Júlia Sampol, Daniel Álvarez Simón, José-María Ramada

**Affiliations:** 1 Servicio de Neumología, Hospital Universitari Vall d’Hebron, 08035 Barcelona, Spain; galo.granados@vhir.org (G.G.); mj.cruz@vhir.org (M.-J.C.); jsampol@vhebron.net (J.S.); danielalvarezsimon@gmail.com (D.Á.S.); 2 Departamento de Medicina, Universitat Autònoma de Barcelona (UAB), 08193 Barcelona, Spain; 3 Centro de Investigación Biomédica en Red, CIBER de Enfermedades Respiratorias (CIBERES), 08036 Barcelona, Spain; 4 Institut Català de Seguretat i Salut Laboral, Departament de Treball, Afers Socials i Famílies, Generalitat de Catalunya, 08019 Barcelona, Spain; santosheca@gmail.com; 5 Centro de Investigación en Salud Laboral, Universitat Pompeu Fabra, 08003 Barcelona, Spain; JRamada@parcdesalutmar.cat; 6 Instituto Hospital del Mar de Investigaciones Médicas (IMIM), Parc de Salut Mar, 08003 Barcelona, Spain; 7 Centro de Investigación Biomédica en Red, CIBER de Epidemiología y Salud Pública (CIBERESP), 08036 Barcelona, Spain

**Keywords:** asbestos, questionnaire, exposure, validation, QEAS-7

## Abstract

Introduction: The seven-item QEAS-7 questionnaire (exposure to asbestos questionnaire) has been designed as a useful and simple tool to establish the probability of exposure to asbestos. The objective of the present study is to validate the QEAS-7 following the recommended methodology. Methods: The QEAS-7 was prospectively administered to 90 subjects with and without asbestos-related disease (ARD), on two consecutive occasions by two independent researchers. Logical and content validity was evaluated by a committee of experts and construct validity through hypothesis testing. Intra- and interobserver reliability was assessed by calculating Cohen’s Kappa index (κ), which was estimated as weak if below 0.40, moderate if between 0.41 and 0.60 and good/very good if above 0.60. The comparison between proportions was examined using Pearson’s Chi-square test. Results: The majority of participants (88.9%) were male. Mean age was 70.8 years (SD = 8.4) and most of the sample had completed primary education but had not progressed further (62.2%). Forty-three had ARD. The logical, content and construct validity of the QEAS-7 was considered adequate both by a committee of experts and by the users interviewed. The mean administration time was 9 min and 25 s (SD = 3 min and 49 s). The verification of the five hypotheses confirmed the construct validity and the intra- and interobserver reliability to be κ = 0.93 and κ = 0.50 respectively. The concordance in the estimation of asbestos exposure was κ = 0.65. Conclusions: The QEAS-7 is a simple, valid and reliable tool for estimating the probability of exposure to asbestos. Its application in clinical practice appears justified. What is already known about this subject? No studies have been published to date on the validation of specific questionnaires designed to determine asbestos exposure for routine use by healthcare staff in the clinical setting. What are the new findings? This questionnaire can be considered a comprehensible, viable, valid and reliable instrument for identifying exposure to asbestos. Its brevity and simplicity of administration make it ideally suited for use in daily clinical practice. How might this impact on policy or clinical practice in the foreseeable future? This questionnaire can be of help for physicians attending to patients with suspected asbestos-related diseases both in the hospital and in the primary care setting.

## 1. Introduction

Asbestos is a fibrous silicate that includes two main types: amphibole, with stiff and pointed fibers, and serpentine, with curved fibers. The most frequently encountered form is chrysotile, a member of the serpentine subgroup [1]. Asbestos was widely used in industry in the twentieth century and has been shown to cause asbestos-related diseases (ARD) such as lung and laryngeal cancer, mesothelioma, asbestosis and other pleural diseases [2]. In Spain, its use was prohibited in 2001; to date 56,373 exposed workers have been recorded in this country [3], although this figure is almost certainly an underestimate.

According to the latest available data, some 125 million people in the world are exposed to asbestos in their workplaces and the number of resulting deaths has been estimated at over 200,000 each year [4,5]. This situation is aggravated by the period of latency between exposure and disease which, depending on the pathology, may vary between 15 and 40 years or more [6]. In the coming decades, then, AD will continue to be diagnosed due to exposures that occurred over the last 30–40 years. Furthermore, thousands of buildings and structures all over the world contain asbestos [7], and their repair, maintenance and demolition pose a clear risk of exposure for workers involved in such tasks.

Diagnosis of ARD requires an accurate knowledge of asbestos exposure. According to expert consensus, exposure is best assessed by consulting an individual’s clinical/occupational history [2], but the fact is that anamnesis also has limitations. In an early study, subjects interviewed could only recall 48% of the jobs they had held, and this percentage decreased with time or when an interviewee reported more than two different jobs [8]. In addition, many physicians know little about the sources of exposure to asbestos, or may overlook sources during patient interview.

In research, one method for establishing exposure to asbestos is the use of structured questionnaires designed and interpreted by hygiene experts [2,9]. Another valid approach is the use of job-exposure matrices which, based on a list of occupations and their coding, allow an estimation of the exposure to different occupational agents such as mineral dust, biological dust and gas-fumes [10,11]. Finally, asbestos detection and analysis methods in lung tissue and bronchoalveolar lavage make it possible to quantify the amount of the mineral deposited in the lung [12,13]; however, these are invasive methods that have not proven to be more efficient than the use of specific questionnaires [14]. 

It should be stressed that the methods just discussed are not applicable in clinical practice, where it is health staff who must record the work history of each patient and where the time available is limited. Clinicians need a viable, valid and reliable tool able to determine asbestos exposure. To the best of our knowledge, no studies have been published to date on the validation of specific questionnaires designed to determine asbestos exposure for routine use by healthcare staff in the clinical setting. The present study aims to validate a semidescriptive questionnaire designed to be easy-to-use and reliable for the identification of asbestos exposure in daily clinical practice.

## 2. Methods

### 2.1. Characteristics of the Questionnaire

The QEAS-7 questionnaire (exposure to asbestos questionnaire) comprises seven questions, three of them designed to establish whether the subject has had occupational exposure to asbestos, two to assess domestic exposure, and two to assess environmental exposure [15]. The questions on occupational exposure are complemented by a list of 48 occupations/activities with risk of exposure to asbestos and a list of 70 materials that might contain this substance (Supplementary Materials). Occupations/activities with the highest risk of asbestos exposure are written in red, and those with lower risk in black. Questions and items of the list of occupations and materials at risk of represent asbestos exposure were chosen by the Expert Committee based on hygienist and clinical expertise. The questionnaire also records demographic data and a work history to identify all the jobs carried out by the respondents, in chronological order, as well as the duration of each job. 

### 2.2. Validation of Questionnaire

#### Study Design and Procedures

This prospective, observational study aimed to validate the QEAS-7 asbestos exposure questionnaire in accordance with well-described procedures [16,17,18]. During 2013 and 2014, a convenience sample was recruited from patients at the outpatient respiratory clinic of a tertiary level public hospital in Barcelona. People of both sexes aged over 18 able to understand Spanish were included and distributed into two groups: patients with and without ARD. The two groups were adjusted according to age, sex and educational level (primary or lower, secondary or higher). All participants received clear and comprehensible information regarding the objectives and scope of the study and were provided with a written summary, and all gave informed consent prior to enrollment. The project was approved by the Ethics Committee of the Vall d’Hebron Hospital with the identification code PR(AG)119/2013. 

The QEAS-7 was administered by an interviewer who was blind to the group to which each subject belonged. Immediately afterwards, participants answered a standardized battery of questions to assess its comprehensibility and applicability (Supplementary Materials). Finally, the interviewer recorded the time taken to administer the questionnaire.

### 2.3. Questionnaire Validation Criteria

#### 2.3.1. Apparent or Logical Validity

After the administration of the QEAS-7, participants answered a standardized battery of questions to assess its comprehensibility and applicability (Supplementary Materials). The Expert Committee empirically evaluated these responses and determined the degree to which, in the opinion of experts and users, the QEAS-7 logically measured what it was intended to measure (i.e., the probability of occupational exposure to asbestos). In accordance with the literature, it was decided that any question that presented difficulty for 15% or more of the participants would be revised [16,19].

#### 2.3.2. Content Validity

The ability of the QEAS-7 to measure the dimensions of the construct was evaluated. The Expert Committee, formed by one expert of occupational medicine and methodology on questionnaire validation, one occupational hygienist, two pneumologists with experience on asbestos related diseases and a biologist, carried out an empirical and formal assessment of the questionnaire, including the analysis of its structure, its dimensions and its adjustment to a conceptual framework that comprises three possible sources of exposure (work, the environment and the home). Particular emphasis was placed on evaluating whether the questionnaire items constituted a representative sample of what was to be measured.

#### 2.3.3. Construct Validity

Known-groups validity analysis techniques were used to test hypotheses and to compare the results obtained when applying the QEAS-7 to groups with a known clinical or demographic status. The following hypotheses (H) were evaluated in relation to the probability of occupational exposure to asbestos:

**Hypothesis** **1** **(H1).** *The questionnaire will confirm definite or probable occupational exposure to asbestos in at least 90% of the participants already known to have ARD. This hypothesis was tested by calculating the absolute number (n) and the proportion (%) of participants who, having been diagnosed with an ARD, were shown by the questionnaire to have had definite or probable occupational exposure.*

**Hypothesis** **2** **(H2).** *The QEAS-7 will detect a higher proportion of definite or probable occupational exposure to asbestos in participants with manual jobs than nonmanual ones. This hypothesis was tested by calculating the absolute number (n) and the percentage (%) of participants in manual and nonmanual jobs with definite or probable occupational exposure.*

**Hypothesis** **3** **(H3).** *The QEAS-7 will detect occupational exposure to asbestos in at least 10% of the participants with no history of exposure in their medical history. The hypothesis was evaluated by calculating the absolute number (n) and the percentage (%) of participants who, without any history of exposure to asbestos in their medical history, presented some degree of occupational exposure according to the questionnaire.*

**Hypothesis** **4** **(H4).** *The QEAS-7 will detect a higher proportion of definite or probable occupational cases of exposure to asbestos among participants known to have an ARD. This hypothesis was tested by calculating the absolute number (n) and the percentage (%) of participants with and without ARD with definite or probable occupational exposure.*

**Hypothesis** **5** **(H5).** *The QEAS-7 will detect a higher proportion of cases of definite or probable occupational exposure to asbestos in participants with pleural plaques and/or asbestos bodies. The hypothesis was tested by calculating the absolute number (n) and the percentage (%) of participants who, after confirmation of the presence of pleural plaques and/or asbestos bodies, were identified as having definite or probable exposure by the questionnaire.*

#### 2.3.4. Intraobserver Reliability or Test–Retest Reliability

Intraobserver reliability or test–retest reliability was evaluated by administering the questionnaire to the same population at two different time points separated by an interval of 2–3 weeks. The questionnaire was administered by the same interviewer, using the same method. This 2–3-week interval meant that the clinical and exposure conditions of the participants were not modified, but that the responses given in the first administration were unlikely to be remembered: thus, the problem of recall bias was avoided.

#### 2.3.5. Interobserver Reliability

Interobserver reliability was evaluated by administering the QEAS-7 to a subgroup of subjects on two occasions, by two independent interviewers, in order to assess the degree of correlation in the responses and the possible influence on these responses by the interviewer.

The probability of occupational exposure to asbestos was independently assessed by two health professionals who were blind to the group to which each participant belonged. One of them was an expert hygienist who knew the QEAS-7 in depth, and the other was a pulmonologist who was not involved in the design of the questionnaire. Using the hygienist’s assessment as a reference, the percentage of false positives and false negatives was estimated and the percentage of over- and underevaluations of exposure was determined.

#### 2.3.6. Criteria for Assessing the Likelihood of Occupational Exposure to Asbestos

Each of the first three questions in the questionnaire was assessed separately to estimate four degrees of likelihood of occupational exposure to asbestos: definite, probable, nonexistent, or unknown. Occupational exposure was deemed to be “definite” when an affirmative answer was given to question 1, on asbestos use in general, and at least one activity or material from the lists was recorded. Likewise, exposure was also estimated to be “definite” when a negative answer was given to question 1 and an affirmative answer to a material or activity with a high risk of exposure from the lists (i.e., those appearing in red).

Occupational exposure was estimated to be “probable” when question 1 was answered affirmatively and when none of the listed activities or materials were recorded. It was also estimated to be “probable” when an affirmative answer was given to a material or activity on the lists with a lower risk of exposure (i.e., those appearing in black).

Occupational exposure was regarded as “nonexistent” when a negative answer to question 1 was recorded together with negative answers to the items on the lists of materials and activities. It was considered “unknown” when the patient answered “don’t know” both to question 1 and to the lists of activities and materials.

### 2.4. Statistical Analysis

The comparison between proportions was analyzed using Pearson’s Chi square test. Test–retest and interobserver reliability were evaluated by calculating Cohen’s Kappa index (κ); they were estimated as weak if κ < 0.40, moderate with κ values between 0.41–0.60, and good or very good if κ > 0.61 [20].

## 3. Results

### 3.1. Sample Characteristics

The validation was performed in 90 participants. Most were men (*n* = 80; 88.9%) with a mean age of 70.8 years (SD = 8.4). With regard to educational level, the largest group (*n* = 56; 62.2%) had completed primary studies but had not progressed further (Table 1). Forty-three had a known diagnosis of ARD, mostly pleural plaques (Table 2).

### 3.2. Criteria for Validating the Questionnaire

#### 3.2.1. Apparent or Logical Validity

Both experts and users regarded the apparent or logical validity of the questionnaire to be adequate. Both considered the questionnaire to be comprehensible, in terms of the wording of its items, and easy to use, given that the mean administration time was 9 min and 25 s (SD = 3 min and 49 s). None of the questionnaire items required reformulation, since none presented difficulties of comprehension to more than 15% of the interviewees.

#### 3.2.2. Content Validity

The experts considered the questionnaire’s content validity to be adequate in terms of its conceptual framework and its capacity to identify the different areas of exposure to asbestos (i.e., occupational, domestic and environmental).

#### 3.2.3. Construct Validity

All five hypotheses tested were confirmed (see Table 3).

#### 3.2.4. Reliability

The test–retest reliability (intraobserver reliability or repeatability) and interobserver reliability showed Cohen’s kappa coefficient values (κ) of 0.93 and 0.50, respectively.

The degree of concordance between the evaluations made by the hygienist (an expert in the use of the questionnaire) and the pulmonologist (a nonexpert) was κ = 0.65. Taking the assessment made by the hygienist as a reference value, a false positive rate of 1.1% and a false negative rate of 2.2% were obtained. The pulmonologist’s assessment was higher than the hygienist’s assessment in 13.2% of cases and lower in 6.6%.

## 4. Discussion

The results of this study show that the QEAS-7 questionnaire can be considered a comprehensible, viable, valid and reliable instrument for identifying exposure to asbestos. Its brevity and simplicity of administration make it ideally suited for use in daily clinical practice.

The QEAS-7’s comprehensibility was demonstrated in this sample of participants with a relatively low educational level (80% had not progressed beyond primary studies). Its ease of application is likely to be an important advantage in clinical practice. The percentage of the general population who have performed jobs at risk of exposure to asbestos is considerable—42% in Europe [21] and 33% in Japan [22]—so the simplicity of administration of the QEAS-7 and the time required (less than 10 min) qualify it as a useful tool in both primary care and specialized services.

The apparent or logical validity and content validity of the QEAS-7 were assessed by an Expert Committee, and the construct validity by using known-groups validity analysis techniques, testing various hypotheses, and focusing only on the probability of occupational exposure to asbestos [16,17]. Five hypotheses were tested in the study, three of them (H1, H4 and H5) related to the sensitivity of the questionnaire (Table 2). The QEAS-7 was able to detect exposure in patients in whom it was known to have occurred, for example in subjects with confirmed ARD (above all in those with pleural plaques). In fact, the confirmation of the third hypothesis, H3, regarding inadvertent exposures, also reflected the questionnaire’s sensitivity. The QEAS-7 indicated exposure to asbestos in 60% of the subjects without known exposure according to previous medical files—a rate far higher than expected, and an indication of the ubiquity of asbestos in the workplace and the lack of knowledge of its presence among the population. Finally, the testing of hypothesis H2 revealed the relationship between manual work and asbestos exposure and may explain the high percentage of exposure in people without ARD detected in our sample. In summary, then, our assessment showed the QEAS-7 to be a valid questionnaire capable of accurately identifying exposure to asbestos.

The results for the questionnaire’s test–retest reliability showed high concordance, indicating a high degree of repeatability. Interobserver reliability was acceptable, with a moderate concordance. Another dimension of reliability, internal consistency, is usually calculated using Cronbach’s alpha coefficient; however, the structure of the QEAS-7 and the scarce interrelation between its items did not allow its application in this case [23].

In the QEAS-7, the likelihood of occupational exposure is determined by the responses to the first three questions. The first question is general and assesses subjects’ knowledge of their previous exposure to asbestos (a standard question in a clinical history interview). Questions 2 and 3, however, are innovative, in that they present lists of activities and materials with a known risk of exposure to asbestos. These lists act as useful prompts, since subjects often have difficulty in remembering specific details regarding their previous occupations. In addition, the distinction made between high-risk and other activities is a great help to the rater assessing the questionnaire. An added value of the QEAS-7 is its ability to detect domestic and environmental exposures to asbestos, i.e., exposures outside the workplace. Although these exposures are much less intense and were not taken into account in the validation of the QEAS-7, in certain cases their detection may be particularly useful [24,25].

The QEAS-7 has instructions for determining the probability of exposure to asbestos. For the purposes of its validation, the expert assessment by a hygienist and the assessment of a pulmonologist not involved in the study design helped to determine the possible existence of biases in the rating of the questionnaire deriving from either the subjectivity or the inexperience of the observer. The concordance between the two assessments was good or very good (k = 0.65), again underlining the usefulness of the QEAS-7 in the healthcare setting. Further studies should analyze its effectiveness specifically in primary care, an area in which the ability to detect asbestos exposure would be extremely valuable.

In addition to assessing the likelihood of exposure to asbestos, the QEAS-7 provides information on the time of the exposure and its duration. Although only the probability of occupational exposure was taken into account in the validation of the questionnaire, the time factor may allow the calculation of the intensity of exposure, even though this assessment is beyond the capacity of general practitioners or pulmonologists without precise knowledge about the use of asbestos in the workplace.

This validation of the QEAS-7 was carried out in the Spanish population, and the determination of the likelihood of exposure to asbestos is limited to this geographical area. In our opinion, however, the QEAS-7 may be a useful tool for determining exposure to asbestos in most industrialized countries, due to their similar patterns of industrial development (and specifically with the regard to the importation of asbestos) over the course of the twentieth century [26,27]. Before the questionnaire can be applied in other countries, however, it needs to be culturally adapted and validated in the target language. The adaptation of QEAS-7 to other countries would also require a review of the lists of products and activities included to reflect the circumstances of the industrial use of asbestos in particular environments, as well as the date of its prohibition.

The present study has some limitations. First, most of the participants had been employed in manual jobs in which they were likely to have been in contact with asbestos, and so their exposure to the substance is probably greater than that of the general population. This raises the possibility of a selection and memory bias that may have overestimated the degree of exposure. However, manual workers are the group with the highest exposure to asbestos and can therefore be considered as the target group for this questionnaire. Second, this validation of the QEAS-7 was carried out in a convenience sample of subjects from an outpatient clinic with and without ARD; therefore, we are unable to assess the applicability and/or usefulness of the questionnaire when administered to employees of firms with very specific risks of exposure to asbestos. In these cases, more exhaustive questionnaires, or questionnaires including specific features, may be necessary.

## 5. Conclusions

In conclusion, this study presents the validation of QEAS-7, a brief questionnaire designed by experts for identification of asbestos exposure in the general population and in routine clinical practice. The results show it to be a simple, comprehensible, valid and reliable tool that can be of great help for physicians attending to patients with suspected ARD both in the hospital and in the primary care setting.

## Figures and Tables

**Table 1:** Description of study participants and results for occupational exposure to asbestos according to the QEAS-7 ***.

	Without ARD * (*n* = 47)		With ARD (*n* = 43)		*p*-Value
Age: mean (SD) **	71.4	(8.2)	70.2	(8.8)	0.497
	n	%	n	%	
Gender					0.412
Male	43	91.5	37	86.0	
Female	4	8.5	6	14.0	
Level of schooling					0.524
Less than primary	13	27.7	6	14.0	
Primary	26	55.3	30	69.8	
Intermediate	4	8.5	4	9.3	
Higher	3	6.5	3	7.0	
Type of work					0.023
Manual	27	57.4	34	79.1	
Nonmanual	20	42.6	9	20.9	
Exposure according to the questionnaire					0.001
Definite	18	38.3	28	65.1	
Probable	5	10.6	9	20.9	
Nonexistent	24	51.1	6	14.0	
Unknown	0		0		

* ARD: asbestos-related disease. ** SD: standard deviation. *** QEAS-7: exposure to asbestos questionnaire (7 items).

**Table 2:** Description of asbestos-related diseases in 43 participants.

Diagnoses	*n*	%
Pleural plaques	22	51.2
Bilateral pachypleuritis	8	18.6
Benign pleural effusion	8	18.6
Mesothelioma	1	2.3
Lung cancer	2	4.7
Asbestosis	6	14.0
Rounded atelectasis	7	16.3
Head and neck carcinoma	1	2.3
Pericardial calcifications	1	2.3

**Table 3:** Tests of the hypotheses for assessing construct validity.

Number	Hypothesis	Results		
H1	At least 90% of participants with ARD will have definite or probable occupational exposure to asbestos according to the questionnaire	Participants with ARD and definite or probable occupational exposure to asbestos		
		*n* = 41; 95%		
H2	The questionnaire will detect a higher proportion of definite or probable occupational exposure to asbestos in participants with manual professions than in those with nonmanual professions	Manual (%)	Nonmanual (%)	*p*-Value
		85%	62.07%	0.015
H3	The questionnaire will detect a higher proportion of definite or probable occupational exposure to asbestos in at least 10% of participants without a history of exposure	Participants without a history of exposure in their medical history and definite exposure		
		*n* = 27; 60%		
H4	The questionnaire will detect a higher proportion of definite or probable occupational exposure to asbestos in participants with ARD	% Patients	% Patients	*p*-Value
		with ARD	without ARD	
		95.35%	61.70%	<0.001
H5	The questionnaire will detect a higher proportion of definite or probable occupational exposure to asbestos in participants with pleural plaques and/or asbestos bodies	% Patients with plaques and/or asbestos bodies	% Patients without plaques and/or asbestos bodies	*p*-Value
		95.83%	63.27%	0.003

ARD: asbestos-related disease.

## Supplementary Materials

The following are available online at https://www.mdpi.com/1660-4601/17/24/9167/s1, Questionnaire S1: Abbreviated asbestos questionnaire.
