# Supplementary material for: Validation of an Asbestos Exposure Questionnaire (QEAS-7) for Clinical Practice
Source: Int J Environ Res Public Health. 2020 Dec 8;17(24):9167. doi: 10.3390/ijerph17249167 (PMC7764759; doi:10.3390/ijerph17249167)
Supplement: Supplementary file 1 [file ijerph-17-09167-s001.pdf]

Abbreviated asbestos questionnaire
Subject's nº: .... Interviewer:..................................
Date of interview:........... /.......... / ....

Patient data

Name and surname(s):

………………………………………………………………………..

Date of birth:…./ ……/……. Age:………….years.

Place of residence (street, number and town/city:

…………………………………………………………………………………………….

Phone (landline): …………………………….
Mobile:…………………………………….

Brief employment history

T.1. Current/most recent place of work (specific activity):…………………………...

Employer: ……………………………………………… Period: ………................

(Please list all your previous jobs. Use as many sheets as necessary to write down all your main occupations)

Previous employment:

T.2. Place of work (specific activity): ………………………….…………….

Employer:…………………………………………….... Period:…………………...

T.3. Place of work (specific activity): ……………………………………….

Employer: …………………………………………….. Period: …………………..

T.4. Place of work (specific activity):………………………………………...

Employer: …………………………………………….. Period: …………………..

T.5. Place of work (specific activity): ……………………………………….

Employer:…………………………………………….. Period:

T.6. Place of work (specific activity):………………………………………...

Employer:…………………………………………….. Period: …………………....

Abbreviated asbestos questionnaire
Subject's nº: .... Interviewer:...................................
Date of interview:........... /.......... / ....

Interview regarding possible exposure to asbestos

1) In any of the jobs you have had, have you ever performed any activities that involved asbestos?

   Yes (please specify) ……………………………………………………………

   No/Don't know

   (If the answer is affirmative for more than one job, check that they are listed in the brief employment history and indicate the reference of the job(s) given an affirmative answer).

2) In any of the jobs you have had, have you ever carried out any of the activities or operations in List A?

   Yes (please specify) ………………..……………………………………………

   No/Don't know

(If the answer is affirmative for more than one job, list each one and indicate the reference in List A)

3) In any of the jobs you have had, have you ever carried out any activities or operations in List B?

   Yes (please specify) ………………..……………………………………………

   No/Don't know

(If the answer is affirmative for more than one job, list each one and indicate the reference in List B)

4) In your home, have you ever had any materials containing asbestos, or any of the materials in List B?

   Yes (please specify) ………………..……………………………………………

   No/Don't know

(If the answer is affirmative for more than one material in the same residence or in more than one residence, list each material and residence, indicating the material containing asbestos or the reference in List B)

Abbreviated asbestos questionnaire

Subject's nº: .... Interviewer:...................................
Date of interview:........... /.......... / ....

5) In your home, has anyone you live with worked with asbestos or carried out any of the activities in List A?

Yes (please specify) ……………….……………………………………………

No/Don't know

(If the answer is affirmative for more than one person living in the same residence, record each person and the activity involving asbestos or the reference in List A)

6) In your home, have you ever carried out any repair or work on materials containing asbestos or any of the materials on list B?

Yes (please specify) ……………….……………………………………………

No/Don't know

(If the answer is affirmative for more than one instance, record each repair involving asbestos or the reference in List B)

7) Do you remember any activities involving asbestos in the neighborhood or near your home, or any of the activities with the reference A.1, A.2, A.3, A.4, A.5, A.6 or A.7 from list A?

Yes (please specify) ……………….……………………………………………

No/Don't know

(If the answer is affirmative for more than one instance, record each activity and indicate the reference in List A)

Abbreviated asbestos questionnaire
Subject's nº: .... Interviewer:..................................
Date of interview:........... /.......... / ....

List A: Activities or lobs of risk

(The ones marked in red are the ones with the greatest likelihood of exposure)

A.1. **Shipyard workers (construction, repair or scrapping of boats)**
A.2. **Workers in warehouses storing construction materials**
A.3. **Sales representatives working with materials containing asbestos (brakes, clutches, thread, rope, cord, fabric, gaskets, cardboard, cardboard gaskets,…)**
A.4. **Manufacturers of friction material (brakes, clutches)**
A.5. **Manufacturers of fiber cement materials (uralite)**
A.6. **Manufacturers of textiles containing asbestos (thread, rope, cord, fabric, gaskets, cardboard, asbestos fabric)**
A.7. **Repairers of friction material (brakes, clutches)**
A.8. Farming (repair or maintenance of vehicles or machines)
A.9. **Workers in insulation or insulating coatings**
A.10. **Masons (installation of fiber cement roofs or covers (plates or tiles)**
A.11. Graphic artists
A.12. Craftsmen and manual and technical activities
A.13. Firefighters
A.14. **Carpenters, formworkers, assemblers or cabinetmakers**
A.15. Drivers who carry out vehicle maintenance
A.16. **Electricians**
A.17. **Workers in production or maintenance of train or subway cars**
A.18. Workers in production or repair of stoves
A.19. Workers in production of paint
A.20. **Plumbers or pipe inspectors**
A.21. **Smelters**
A.22. Workers in the food and beverage industry (repair or maintenance)
A.23. Workers in ceramics (repair or maintenance)
A.24. Workers in the plastic or rubber industry (repair or maintenance)
A.25. Workers in the glass industry
A.26. Kitchen installers
A.27. Jewelers
A.28. Chimney sweeps
A.29. Workers in rehabilitation, repair or maintenance of industrial buildings
A.30. **Workers in rehabilitation, repair or maintenance of fiber cement materials (uralite)**
A.31. **Car mechanics**
A.32. **Elevator mechanics**
A.33. **Mechanics in industry**
A.34. **Workers in asbestos mines**
A.35. **Dock workers**
A.36. Painters of buildings
A.37. Workers repairing industrial machines
A.38. **Workers in the repair or maintenance of municipal water distribution networks**
A.39. **Workers repairing electric motors**
A.40. Workers repairing and painting of car bodies
A.41. **Boiler room workers**
A.42. Workers in gas, nuclear and power plant services
A.43. Military service and armed forces (repair or maintenance)
A.44. **Welders (also brass and tin makers)**
A.45. Ragpickers
A.46. **Roofers. Installation or repair of roofs or facades**
A.47. **Ventilation technicians and air conditioning installers**
A.48. Textile workers (heat-resistant clothing)

Abbreviated asbestos questionnaire
Subject's nº: .... Interviewer:...................................
Date of interview:........... /.......... / ....

List B: Materials that contain asbestos (MCA)

(This list is not exhaustive; other products and materials may be added)

MCA in construction materials

The most widely used asbestos material is fiber cement or asbestos-cement, which has been applied in the construction industry in the form of:

B.1. Corrugated plates on roofs and rain walls
B.2. Flat plates on facades (rain walls) and balconies
B.3. High pressure water pipes
B.4. Drainpipes
B.5. Water tanks
B.6. Plant pots
B.7. Exhaust ducts, chimneys and shunts
B.8. Air conditioning ducts
B.9. Fences for gardens and livestock
B.10. Benches and outdoor tables

Other forms of presentation of asbestos in construction are:

B.11. Coating for the protection of metallic structures and as thermo-acoustic coating
B.12. Asbestos fibers, used as interior insulation of fire doors in air chambers in walls and ceilings
B.13. Acoustic and thermal insulation
B.14. Mortars for the protection of metallic structures
B.15. Plates of varying densities for soundproofing
B.16. Ceiling plates
B.17. Mixed with paint and putty, it was used as a thixotropant; it also provided flame-retardant characteristics.
B.18. Mixed with plastic material for vinyl flooring
B.19. Mixed with other materials imitating wood, in interior decoration
B.20. Adhesives and glues

MCA in machines and installations

As a **textile** material, asbestos may be found in:

B.21. Blankets and felts for lining pipes
B.22. Braids used to wrap pipes in order to isolate them
B.23. Flame retardant fabrics for fire protection. Blankets, theater curtains, clothes
B.24. Asbestos thread and cord
B.25. Industrial and commercial felts
B.26. Electrical insulation material
B.27. Thermal protective clothing: gloves, clothing, aprons, etc.
B.28. Gaskets

As **cardboard, asbestos** can be found in:

B.29. Cardboard or low-density plates to protect metallic structures or sources of heat (kitchens, fireplaces, etc.)
B.30. Air conditioning ducts
B.31. Flame-retardant absorbent paper
B.32. Catalytic filters and insulation devices in heating appliances that use liquefied gas
B.33. Protection for handling molten glass
B.34. Corrosive gas and vapor lines
B.35. Anti-heat and flame retardant components
B.36. Insulating products

Abbreviated asbestos questionnaire

Subject's nº: .... Interviewer:..................................

Date of interview:........... /.......... / ....

B.37. Coating of electric motors to protect them from sources of heat
B.38. Sealing gaskets

As **friction material**

B.39. Brake pads
B.40. Drum brakes
B.41. Clutches/transmission components
B.42. Industrial friction material

Other applications:

B.43. Wire drawing
B.44. Coating of glassware clamps
B.45. Filters for beverages (beer and wine) and oils
B.46. Filters for the transportation, distribution and use of gas
B.47. Coating of clamps for crucibles
B.48. Thermal insulation in distillation columns
B.49. Road surfacing material
B.50. Fixing and joints for tiles
B.51. Plastics and rubbers reinforced with asbestos
B.52. Plastic components for electric motors
B.53. Plastic components for molded products
B.54. Sealing gaskets
B.55. Electrical insulation parts with resins
B.56. Filling of acetylene bottles

MCA used in the household

B.57. Thermal plate stoves
B.58. Electric resistance stoves
B.59. Kitchens with electric heating elements
B.60. Electric heaters
B.61. Butane gas heaters
B.62. Ironing board covers
B.63. Hair, feet and hand dryers
B.64. Toasters
B.65. Heat-resistant oven gloves
B.66. Heat protection for formica furniture below ovens

MCA in other applications or uses in laboratories (companies, institutes, technical schools, universities)

B.67. Grids for heating glass appliances
B.68. Internal insulation linings of muffle stoves and furnaces
B.69. Pincers to hold crucibles

Abbreviated asbestos questionnaire

Subject's nº: .... Interviewer:...................................

Date of interview:........... /.......... / ....

OBSERVATIONS ON THE PROCEDURE FOR THE USE OF THE QUESTIONNAIRE

The interview will start with the recording of general data, i.e., identification of the subject, the interviewer, the date on which the interview is carried out, and patient's identification data.

A brief employment history will be compiled, listing the names of the last company where the subject worked, and also all previous companies. The period of time the subject spent at each of the companies will be specified in years (e.g., 1990-1995).

After listing the subject's jobs or occupations, an interview will be conducted regarding the possible exposure to asbestos.

First question

Affirmative answer: no further questions are required. Interviewers should check that the job(s) with affirmative answer(s) are listed in the brief employment history and the corresponding reference(s) will be recorded (T.1, T.2….).

To check the accuracy of the first answer, interviewers may ask questions 2 and 3; they should apply the same criteria at all times.

Negative answer or "don't know": the interviewer asks the second question.

Second question

Affirmative answer: no further questions are required. The reference(s) of the activities or jobs in List A will be noted.

Negative answer or "don't know": the interviewer asks the third question.

Third question

Affirmative answer: no further questions are required. The reference(s) of the materials in List B will be noted.

Negative answer or "don't know": the interviewer administers the questionnaire regarding possible exposure to asbestos

An affirmative answer to any of the first three questions suggests that the interviewee has had occupational exposure to asbestos. Since the scale of occupational exposure to asbestos is much higher than the possible domestic and environmental exposures, there is no need to continue the interview; the exposure to asbestos will be classified as occupational.
If the interview proceeds, the following three questions will be asked to determine possible domestic exposure to asbestos.
Regardless of the answers, the interview will continue with the seventh question to determine the interviewee's possible environmental exposure to asbestos.
